# Probabilistic model data of time-dependent accident scenarios for a mixing tank mechanical system

**DOI:** 10.1016/j.dib.2019.104243

**Published:** 2019-07-08

**Authors:** Alessandro Mancuso, Michele Compare, Ahti Salo, Enrico Zio

**Affiliations:** a Department of Mathematics and Systems Analysis, Aalto University, Finland; b Department of Energy Engineering, Politecnico di Milano, Italy; c Aramis s.r.l, Milano, Italy; d MINES ParisTech, PSL Research University, CRC, Sophia Antipolis, France

**Keywords:** Risk analysis, System reliability, Preventive safety measures, Dynamic bayesian networks, Portfolio optimization

## Abstract

This article presents the risk assessment of a mixing tank mechanical system based on the failure probabilities of the components. Possible component failures can cause accidents which evolve over multiple time stages and can lead to system failure. The consequences of these accident scenarios are analyzed by quantifying the failure probabilities and severity of their outcomes. Illustrative costs and updated failure probabilities are provided to evaluate preventive safety measures. Data refers to the results of the Bayesian model presented in our research article (Mancuso et al., 2019).

**Table:** Specifications table

Subject	Safety, Risk, Reliability and Quality
Specific subject area	Portfolio optimization for risk mitigation
Type of data	Tables
How data were acquired	Analysis of the numerical results of the Bayesian model [1]
Data format	Analyzed data
Parameters for data collection	Journal reputation
Description of data collection	Literature review
Data source location	Institution: Aalto University City: Helsinki Country: Finland
Data accessibility	With the article
Related research article	Mancuso, A., Compare, M., Salo, A. and Zio, E., 2019. Portfolio optimization of safety measures for the prevention of time-dependent accident scenarios. Reliability Engineering & System Safety, 190 (106500). DOI: https://doi.org/10.1016/j.ress.2019.106500

**Table:** 

**Value of the data** • The failure probabilities of the components of a mixing tank mechanical system can be used for benchmarking in future research. • Examples of conditional probability tables illustrate the modelling of time-dependent accident scenarios. • Novel applications for probabilistic risk assessment are possible based on the data in this article.

## 1 Data

This article presents the probabilistic model data of the time-dependent accident scenarios for a mixing tank mechanical system. Specifically, we revisit the earlier analyses of the accident scenarios by Khakzad et al. [2] to illustrate the methodology presented in our research article [1]. One of such accident scenarios occurred on 14 June 2006 at Universal Form Clamp in Bellwood (Illinois, U.S.) through a vapor cloud ignition [3].

Table 1 shows the failure probabilities of *Alarm* and *Sprinkler* for different ways of activating such components during an accident. In particular, the activation occurs if the vapor is ignited or if there is a specific amount of vapor concentration in the air, even though the vapor is not ignited.

**Table 1:** Conditional probabilities of *Alarm* and *Sprinkler* at τ=0 (τ refers to the time stage of the Bayesian model).

	Vapor	Controlled		Overflow	
	Ignition	No spark	Spark	No spark	Spark
Alarm	Activation	0	0	0.7750	0.9987
	No activation	1	1	0.2250	0.0013
Sprinkler	Activation	0	0	0.70	0.96
	No activation	1	1	0.30	0.04

Based on the analyses by Khakzad et al. [2], Table 2 lists the system components and their failure probabilities. In addition, we assume that the activation of *Sprinkler* reduces the probability of delayed ignitions by 50%, as detailed in Table 3 (last row, first and second columns). For this reason, the activation of the *Sprinkler* for a vapor concentration in the air could prevent delayed ignitions.

**Table 2:** List of components and respective failure probability.

Component	Symbol	Failure probability
Sensor	Sensor	0.0400
Pneumatic unit	P_unit	0.2015
Temperature control system	T_ctrl_sys	OR gate
Operator	Operator	0.0200
Infrared thermometer	Thermo	0.0468
Temperature measurement system	T_sys	OR gate
Manual steam valve	M_valve	0.0243
Automatic steam valve	A_valve	0.0276
Automatic temperature control system	ATCS	OR gate
Manual temperature control system	MTCS	OR gate
High temperature protection system	HTPS	AND gate
Ventilation	Vent	0.0150
Fan	Fan	0.0100
Belt	Belt	0.0500
Duct	Duct	0.0010
Ventilation system	Vent_sys	OR gate
Vapor overflow	Vapor	AND gate
Ignition barrier	Ignition	0.1000
Water sprinkler system	Sprinkler	0.0400, 0.3000
Alarm system	Alarm	0.0013, 0.2250

**Table 3:** Conditional probabilities of *Ignition* at τ>0 (τ refers to the time stage of the Bayesian model).

	Ignition [τ−1]	No spark		Spark	
	Sprinkler [τ−1]	Activation	No activation	Activation	No activation
Ignition [τ]	No spark	0.95	0.9	0	0
	Spark	0.05	0.1	1	1

Table 4 lists the nine possible outcomes of the accident scenarios where the state *Safe* represents the outcome following the non-occurrence of the system failure (*Vapor = Controlled*). The other outcomes are caused by malfunctions of some system components. Due to the activation of *Sprinkler*, accident consequences $C_{1}$ and $C_{2}$ are less severe than $C_{3}$ and $C_{4}$, respectively. This information is helpful in eliciting the disutility functions to specify the ranking of the outcome severity. The last column of Table 4 shows illustrative disutility values that quantify the severity of the outcomes.

**Table 4:** List of accident outcomes (C refers to the accident consequences, numbered based on increasing severity).

Outcome	Symbol	Disutility
Controlled vapor	*Safe*	0
Safe evacuation	$C_{1}$	10
Wet vapor cloud near the ground	$C_{2}$	15
Safe evacuation with possibility of delayed ignition	$C_{3}$	30
Vapor cloud with possibility of delayed ignition	$C_{4}$	40
Fire, moderate property damage, low death toll	$C_{5}$	60
Fire, high property damage, low death toll	$C_{6}$	80
Fire, moderate property damage, high death toll	$C_{7}$	90
Fire, high property damage, high death toll	$C_{8}$	100

Based on the failure probabilities in Table 2, the Bayesian model computes the occurrence probabilities of the outcomes of the accident scenarios, reported in Table 5 for each time stage. The deployment of preventive safety measures on some selected components mitigates the risk of the negative outcomes. Table 6 lists the alternative preventive safety measures (second column) that affect the occurrence of failures of specific components (first column). The last two columns of Table 6 report illustrative costs and updated failure probabilities of the components. In particular, the preventive safety measure *Synergy* refers to a combination of *Calibration test* and *Sensor*: if both measures are installed, this synergy effect yields more benefits than installing independent measures. The updated failure probabilities of *Sprinkler* and *Alarm* refer to the two different failure scenarios detailed in Table 1.

**Table 5:** Probabilities of accident outcomes at each time stage (C refers to the accident consequences).

Outcome	τ=0	τ=1	τ=2	τ=3	τ−=4	τ=5
*Safe*	0.998319	0.998319	0.998319	0.998319	0.998319	0.998319
$C_{1}$	0.000820	0.001226	0.001289	0.001256	0.001202	0.001144
$C_{2}$	0.000238	6.539252e-05	1.485681e-05	3.229053e-06	6.934547e-07	1.484231e-07
$C_{3}$	0.000352	0.000116	3.270228e-05	8.908458e-06	2.410073e-06	6.510108e-07
$C_{4}$	0.000102	6.202325e-06	3.767917e-07	2.289007e-08	1.390572e-09	8.447723e-11
$C_{5}$	0.000161	0.000264	0.000343	0.000411	0.000475	0.000536
$C_{6}$	6.713624e-06	2.083401e-06	5.733853e-07	1.552510e-07	4.193539e-08	1.132327e-08
$C_{7}$	2.097377e-07	2.850967e-08	5.062283e-09	1.019337e-09	2.140727e-10	4.552654e-11
$C_{8}$	8.739072e-09	5.313530e-10	3.227972e-11	1.960993e-12	1.191303e-13	7.237167e-15

**Table 6:** List of preventive safety measures and respective failure probability.

Component	Preventive safety measure	Cost [k€]	Failure probability
P_unit	Inspection plan	60	0.1500
	Duplication	80	0.100
M_valve	Calibration test	30	0.0200
	Sensor	40	0.0150
	Synergy	60	0.0100
A_valve	Calibration test	30	0.0200
	Sensor	40	0.0150
	Synergy	60	0.0100
Belt	Periodic test	40	0.0300
	Condition monitoring	100	0.0100
Ignition	Tank blanketing	70	0.0800
	Inerting systems	100	0.0600
	Hypoxic air technology	150	0.0400
Sprinkler	Standard response	40	0.0300, 0.2000
	Quick response	80	0.0100,0.1000
Alarm	Semi-conductor sensor	60	0.0013, 0.2000
	Catalytic gas sensor	80	0.0013, 0.1500
	Electrochemical cells	100	0.0013, 0.1000

## 2 Experimental design, materials, and methods

The failure probabilities of the components in Table 2 are provided by the article by Khakzad et al. [2]. Gates represents logic structures of the Bayesian model in our research article [1]. The failure probabilities in Table 6 have been obtained by reducing the initial failure probability of the components, based on a specific reduction rate for each preventive safety measure. These values illustrate the viability of the Bayesian model [1], but do not represent any actual system. The occurrence probabilities of the outcomes of the accident scenarios have been computed by GeNIe Modeler [4] through the Dynamic Bayesian Network presented in our research article [1]. Finally, the severity of the outcomes has been quantified through the trade-off weighing approach SWING [5].
